# Coping experiences of trans women throughout their gender-affirmation

**DOI:** 10.1192/j.eurpsy.2025.366

**Published:** 2025-08-26

**Authors:** G. Yıldız Aytaç, D. Hiçdurmaz, K. Başar

**Affiliations:** 1 Psychiatric Nursing Department, Hacettepe University Faculty of Nursing; 2 Department of Psychiatry, Hacettepe University Faculty of Medicine, Ankara, Türkiye

## Abstract

**Introduction:**

Trans women can encounter various struggles throughout their gender-affirmation. There is a need for further understanding of trans women’s experiences to gain deeper insights into how they cope throughout this process. The development of psychosocial support services that are adapted to their personal needs is crucial to enhancing their coping strategies.

**Objectives:**

The current study aimed to examine the coping experiences of trans women throughout their gender-affirmation.

**Methods:**

This qualitative descriptive study utilized in-person, semi-structured interviews with 12 trans women to gather in-depth data on their coping experiences. Content analysis was employed to analyze the data.

**Results:**

The experiences of trans women emerged in five themes. Four themes correspond to four distinct phases: “self-discovery,” “self-acceptance,” “coming out to others,” and “after coming out to others,” each characterized by its own coping mechanisms. The fifth theme was labeled “to facilitate coping…”. Trans women have a heightened need for support during the periods “when they confront the possibility that their situation will not change,” “when they accept themselves but attempt to decide how they can move forward in life,” and “when they first come out to people around them.” The study indicates the critical role of addressing family and social stigma in trans women’s coping throughout their gender-affirmation. Furthermore, the study unveils a striking finding that efforts to facilitate trans women’s coping throughout their gender-affirmation extend beyond the purview of mental health professionals. It reveals that these efforts have dimensions that concern the entire healthcare system, the legal system, social security, labor, and working conditions.

**Conclusions:**

The study highlights the importance of psychosocial support and improved access to these services to bolster trans women’s coping mechanisms throughout their gender-affirmation, with particular emphasis on the specific periods identified above. The psychosocial support for trans women should encompass not only them but also extend to their families, significant others, and the community in which they live, adopting a holistic approach.

**Disclosure of Interest:**

None Declared

